# A comparative analysis of health technology assessment decision-making for cancer drugs in Germany and England

**DOI:** 10.3389/fpubh.2026.1733239

**Published:** 2026-03-17

**Authors:** Jiaqi He, Haibo Wei, Qihui Mo, Yanxi Liu, Xinyu Li, Yue Yang, Lijuan Tian

**Affiliations:** 1 School of Business Administration, Shenyang Pharmaceutical University, Shenyang, China; 2 School of Pharmaceutical Sciences, Tsinghua University, Beijing, China; 3 School of Biomedical Engineering, Hainan University, Sanya, China; 4 College of Letters and Science, University of California, Davis, Davis, CA, United States; 5 College of Pharmaceutical Sciences, Soochow University, Suzhou, China

**Keywords:** cancer drugs, health technology assessment, decision-making, G-BA, NICE

## Abstract

**Introduction:**

The rising cost of cancer drugs places a heavy burden on patients and public healthcare systems, posing an ongoing challenge of balancing access with efficient resource allocation. Health technology assessment (HTA) serves as a critical basis for reimbursement decisions, evaluating both clinical and economic value. Although the Federal Joint Committee (G-BA) and the National Institute for Health and Care Excellence (NICE) have established standardized HTA frameworks, differences in their assessment methods and decision-making processes may lead to divergent outcomes. This study compares key factors influencing cancer drug assessments by G-BA and NICE and identifies drivers of discrepancies between the two agencies.

**Methods:**

We reviewed 171 matched drug-indication pairs of cancer drugs assessed by G-BA and NICE between 1 January 2020 and 1 June 2025. Logistic regression identified key factors influencing outcomes, while decision tree models explored pathways through which these factors shaped results. The first regression further examined determinants of discordant HTA outcomes between the two agencies.

**Results:**

Among analyzed drugs, NICE issued positive recommendations in 90.64% of cases, whereas G-BA granted an additional benefit in only 59.06%. G-BA outcomes were significantly associated with improvements in mortality (*P* = 0.018), side effects (*P* = 0.043), and orphan drug designation (*P* < 0.0001). NICE recommendations were primarily influenced by low risk of bias (*P* = 0.028) and incremental cost-effectiveness ratios (ICERs) ≤ £30,000 per QALY (*P* = 0.054). Concordant HTA outcomes between G-BA and NICE were more likely when evidence was derived from randomized controlled trials (RCTs; *P* = 0.0003), demonstrated a low risk of bias (*P* = 0.003), or showed improvements in mortality (*P* = 0.003) and morbidity (*P* = 0.009).

**Conclusion:**

Despite differences in evaluation frameworks and institutional settings across countries, high-quality clinical evidence remains the central foundation for concordant HTA decisions. The findings provide policy-relevant insights for optimizing reimbursement policies and improving the efficiency of healthcare resource allocation. Moreover, they offer important implications for policymakers and manufacturers by clarifying the key drivers of HTA decision-making.

## Introduction

The high cost of cancer drugs imposes a substantial economic burden on patients and public healthcare systems (1, 2). While ensuring patients access to innovative treatments, effectively containing healthcare expenditures has become a central objective of health policy worldwide. Health Technology Assessment (HTA) plays a crucial role in evaluating healthcare interventions and informing decision-making (3). HTA outcomes provide evidence-based support for healthcare resource allocation, including decisions on pricing and reimbursement for health technologies (4).

In practice, HTA primarily relies on two core methodological approaches. Firstly, involving systematic evaluations of the clinical effectiveness of health technologies based on the principles of evidence-based medicine (EBM). Secondly, assessing the efficiency of healthcare resource allocation through cost-effectiveness analysis (CEA) and its variants (5). Germany and England have both established standardized HTA appraisal procedures (6), representing two distinct institutional models centered on additional benefit assessment and cost-effectiveness analysis, respectively. Nevertheless, differences persist between the two countries in terms of HTA evaluation dimensions and outcomes. Prior research indicates that the differences in HTA outcomes among European agencies are primarily attributable to differences in legal framework, institutional arrangements, and assessment criteria (7).

In England, the National Institute for Health and Care Excellence (NICE) evaluates the clinical effectiveness and cost-effectiveness of newly approved therapies and issues recommendations regarding reimbursement within the National Health Service (NHS) (8). NICE's recommendations are highly informed by incremental cost-effectiveness ratios (ICERs), with an implicit threshold of £20,000 to £30,000 per quality-adjusted life year (QALY) (9). For treatments targeting end-stage or highly severe diseases, NICE applies weighting factors of 1.2 and 1.7 to QALYs, thereby adjusting the cost-effectiveness threshold (10). Additionally, the NHS has established the Cancer Drug Fund (CDF) to provide temporary funding for cancer drugs with promising but uncertain clinical or economic evidence (11). Under this mechanism, NICE may issue conditional recommendations, allowing restricted use while additional evidence is generated to support subsequent reappraisal (12).

Germany formally implemented the Arzneimittelmarkt-Neuordnungsgesetz (AMNOG) in 2011, which mandates an early benefit assessment for new active substances or new therapeutic indications (13). The objective is to determine whether sufficient evidence demonstrates additional medical benefits compared to appropriate comparator therapies. Manufacturers are required to submit evidence to The Federal Joint Committee (G-BA) on patient-relevant outcomes, including mortality, morbidity, and health-related quality of life (HRQoL). G-BA then determines the presence and magnitude of additional benefit (14, 15). In contrast to NICE, which primarily evaluates cost-effectiveness using the incremental cost per QALY gained, G-BA focuses on the existence, magnitude, and therapeutic relevance of additional benefit relative to the appropriate comparator therapy. The assessment outcome directly influence subsequent price negotiations (16). If an additional benefit is confirmed, reimbursement prices are negotiated between the National Association of Statutory Health Insurance Funds and the manufacturer. If no agreement is reached, the final price is determined by an independent arbitration board (17). If no additional benefit is established, the drug is subject to reference pricing (18).

Previous studies have mainly examined the relationship between HTA outcomes and factors such as annual treatment costs or budget impact. However, limited attention has been given to examining, from an institutional perspective, the divergent conclusions reached by different HTA frameworks when evaluating identical drug-indication combinations, nor the factors influencing these differences. Therefore, we systematically compare between G-BA and NICE for identical drug-indication pairs. It examines key determinants of HTA outcomes for cancer drugs under different institutional logics, thereby elucidating how divergent decision-making mechanisms emerge across HTA systems.

## Methods

### Sample selection

We collected data from the official websites of G-BA and NICE, including completed HTAs (19, 20). The study covered cancer drug evaluations conducted between 1 January 2020 and 1 June 2025. Only single-indication assessments were included and subsequently matched into drug-indication pairs. Based on previous research, we also identified variables that may influence assessment outcomes and collected corresponding data.

### Data extraction

Data were sourced from appraisal reports published on the official websites of the G-BA and NICE, from which the following characteristics were extracted: study design, risk of bias in clinical trials, clinical outcome measures, patient cohort size, daily treatment cost per patient, ICER values reported by NICE, and the final assessment outcomes from both G-BA and NICE.

Regarding clinical endpoints, G-BA assessment documents reported patient-relevant outcomes, including mortality, morbidity, quality of life (QoL), and side effects, whereas NICE appraisal documents reported endpoints such as overall survival (OS), progression-free survival (PFS), and QoL. Risk of bias (RoB) variables were extracted from the final determinations reported in official G-BA and NICE appraisal documents. No independent reassessment of the original clinical trials was undertaken by the authors. This variable primarily reflects trial-level internal validity (e.g., randomization, blinding, and handling of loss to follow-up), and excludes uncertainty related to economic modeling.

Consistent with previous studies, daily treatment costs were calculated based on the total dose required for a complete treatment cycle (21, 22). When both the initial and maintenance doses were reported within the same cycle, maintenance doses were used. Patient numbers were estimated by averaging the minimum and maximum values reported in appraisal documents.

### Statistical analysis

To examine factors influencing evidence consideration and HTA outcomes under different frameworks, we analyzed determinants of G-BA and NICE HTA outcomes from two perspectives: (i) all matched drug-indication pairs and (ii) pairs with discordant HTA outcomes. A matched pair was defined as assessments of the same active ingredient for comparable therapeutic indications and target populations.

Matching followed Schaefer's methodology (23), incorporating matching analyses only when different agencies assessed the same primary therapeutic indication or clinically comparable indications. Given potential variations among HTA bodies in patient subgroup stratification, choice of comparator treatments, and population definitions, comparability was defined as assessments of the same active ingredient conducted under consistent disease types, treatment lines, and core population characteristics. Matching was determined based on the technology's primary therapeutic indication and the clearly defined target population in the assessment documentation. Furthermore, consistent with prior studies, when multiple patient subgroups exist within a primary therapeutic indication, this study selected the subgroup with the most favorable HTA outcome for the matching analysis. This minimized incomparability bias arising from divergent subgroup delineations (24). All matching procedures were independently conducted by two researchers according to pre-established criteria. Discrepancies were resolved through discussion and adjudication by a third researcher to ensure the objectivity and methodological reproducibility of matching outcomes.

For statistical analysis, we used a combined approach of logistic regression and rpart decision tree modeling for all matched pairs. First, univariate and multivariate logistic regression analyses were performed to identify key determinants of HTA outcomes. Subsequently, Classification and Regression Trees (CART) models were constructed using the rpart package. At each node, the Gini index served as the splitting criterion, selecting variables that maximized node purity to build a hierarchical decision path structure. Unlike the pre-determined consideration sequence in the actual G-BA and NICE assessment processes, the decision tree model determines variable splitting order based on data-driven optimal splitting principles. This approach explores the pathway effects of key factors and their combinations on health technology assessment outcomes. For matched pairs with discordant results between the two countries, Firth's penalized logistic regression was applied to correct for potential bias arising from small sample sizes or rare events. This method corrects biased estimates in small samples or rare events by penalizing likelihood, thereby quantifying the independent effects of predictor variables on the probability of inconsistency between G-BA and NICE outcomes. It also examines differences in the effects of relevant factors across the two countries' assessment systems.

To ensure model stability, we required at least five observations per outcome category within each indication level. Given the limited sample size and potential for complete separation, Bayesian generalized linear modeling (bayesglm) with weakly informative priors was used to enhance estimation stability relative to conventional logistic regression. Furthermore, prior to multivariate modeling, univariate analyses were conducted for each predictor to screen for unstable estimators, thereby informing variable inclusion and robustness control.

Additionally, because the categorical variables lacked a natural order or equidistant levels, dummy variables were used in the regression analysis. The reference categories for each categorical variable were defined as follows: bias risk (High), Mortality (Negative), Morbidity (Negative), QoL (Negative), Side effects (Negative), OS (No significant improvement), PFS (No significant improvement), ICER (≤ 20,000 per QALY), Orphan drug (No), RCT (No), Indication (Breast cancer). Effect estimates for each categorical level were interpreted relative to the corresponding reference category.

All statistical analyses were conducted in *R* Studio. *P*-values less than 0.05 were considered statistically significant.

## Results

**Figure 1:**
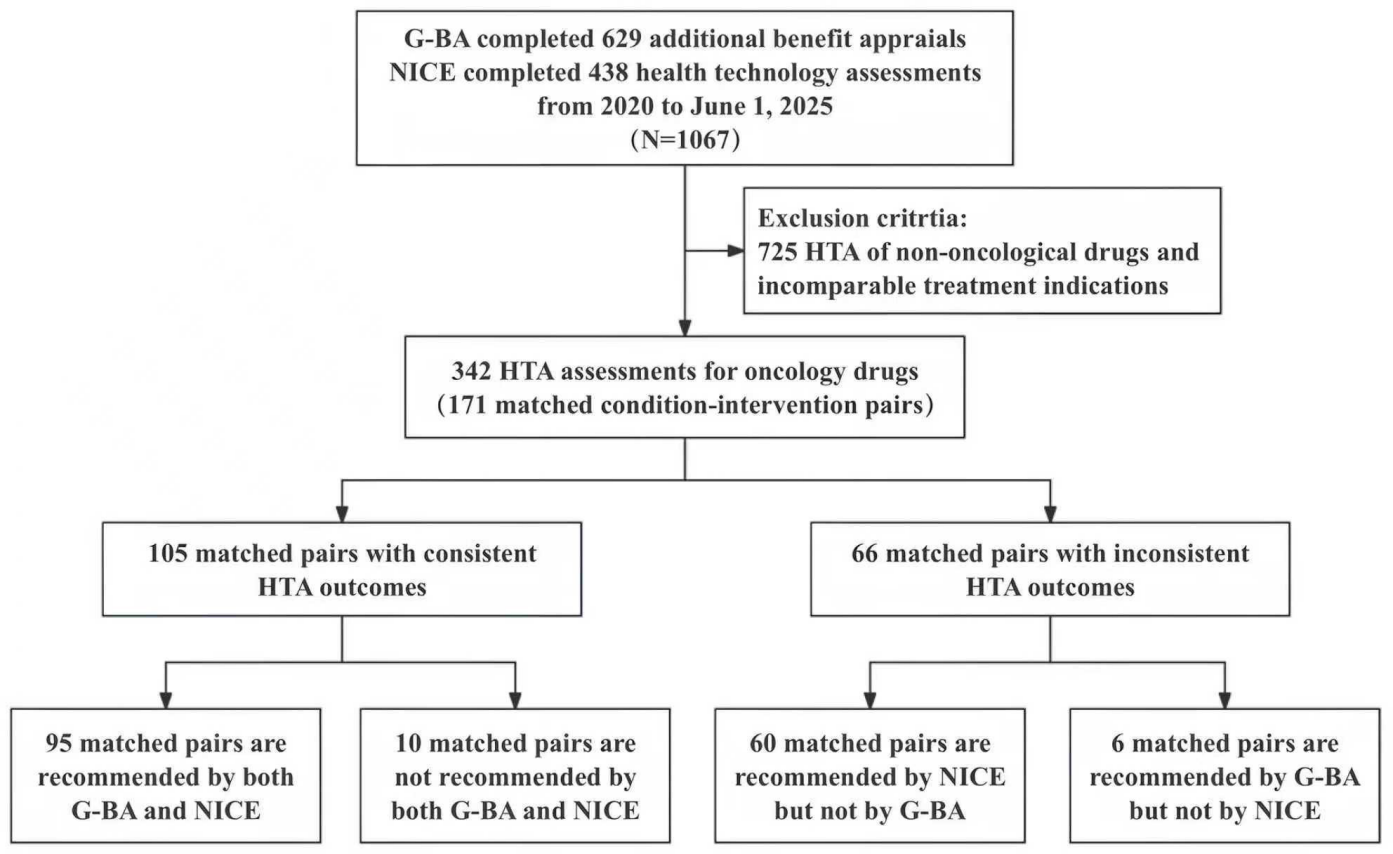
The framework of inclusion for cancer drugs.

Between January 1, 2020, and June 1, 2025, G-BA completed 629 additional benefit assessments, whereas NICE finalized 438 health technology assessments (HTAs). From these, 342 HTAs (171 matched drug-indication pairs) evaluating identical cancer drugs for comparable indications across both agencies were identified (Figure 1). Breast cancer represented the most frequent indication (n = 23, 13.45%), followed by non-small cell lung cancer (n = 22, 12.87%) and lymphoma (n = 21, 12.28%), with 40 pairs (23.39%) involving orphan drugs. Randomized controlled trials informed 138 G-BA assessments (80.70%) and 131 NICE evaluations (76.61%). Most assessments were published between 2021 and 2025, including 118 G-BA (69.01%) and 119 NICE (69.59%) reports. Low-risk-of-bias ratings were assigned to 129 G-BA (75.44%) assessments, compared with 92 NICE (53.80%) assessments (Table 1).

**Table 1:** Descriptive statistics for G-BA decisions and NICE recommendations.

**Variable**	**G-BA decision**		**NICE recommendation**	
	**Additional benefit**, ***N*** = **101 (%)**	**No additional benefit**, ***N*** = **70 (%)**	**Recommended**, ***N*** = **155 (%)**	**Not recommended**, ***N*** = **16 (%)**
**Orphan drug status**				
No	67 (66.34%)	64 (91.43%)	119 (76.77%)	12 (75.00%)
Yes	34 (33.66%)	6 (8.57%)	36 (23.23%)	4 (25.00%)
**Clinical study evidence: randomized-controlled trial (included in a systematic review or meta-analysis)**				
Not available	9 (9.78%)	24 (34.29%)	36 (23.23%)	4 (25.00%)
Available	92 (90.22%)	46 (65.71%)	119 (76.77%)	12 (75.00%)
**Risk of bias**				
Not available	7 (6.93%)	19 (27.14%)	22 (14.19%)	3 (18.75%)
High	4 (3.96%)	12 (17.14%)	7 (4.52%)	4 (25.00%)
Medium	0 (0.00%)	0 (0.00%)	40 (25.81%)	3 (18.75%)
Low	90 (89.11%)	39 (55.72%)	86 (55.48%)	6 (37.50%)
**Publication year**				
2020 and before	39 (38.61%)	14 (20.00%)	49 (31.61%)	3 (18.75%)
2021–2025 (January–June)	62 (61.39%)	56 (80.00%)	106 (68.39%)	13 (81.25%)
**Drug indication**				
Breast cancer	21 (20.79%)	2 (2.86%)	22 (14.19%)	1 (6.25%)
Non-small cell lung cancer	9 (8.91%)	13 (18.57%)	18 (11.61%)	4 (25.00%)
Lymphoma	9 (8.91%)	12 (17.14%)	19 (12.26%)	2 (12.50%)
Leukemia	14 (13.86%)	6 (8.57%)	20 (12.90%)	0 (0.00%)
Multiple myeloma	5 (4.95%)	4 (5.71%)	9 (5.81%)	0 (0.00%)
Melanoma	5 (4.95%)	4 (5.71%)	9 (5.81%)	0 (0.00%)
Prostate cancer	5 (4.95%)	4 (5.71%)	7 (4.52%)	2 (12.50%)
Renal cell carcinoma	5 (4.95%)	4 (5.71%)	8 (5.16%)	1 (6.25%)
Gastric cancer	4 (3.96%)	2 (2.86%)	5 (3.23%)	1 (6.25%)
Others	24 (23.76%)	19 (27.14%)	38 (24.52%)	5 (31.25%)

Among the 171 matched pairs, G-BA found that 101 interventions (59.06%) had additional benefit, while 70 (40.94%) had none. In comparison, NICE recommended 133 treatments (77.78%), conditionally recommended 22 (12.86%), and did not recommend 16 (9.36%), of which six approvals (five recommended and one conditional) were granted through the cancer drugs fund (CDF).

### Analysis of key factors influencing health technology assessment outcomes

#### Key factors influencing the outcomes of G-BA health technology assessments

The multivariate logistic regression revealed that improvements in mortality and side effects, relative to appropriate comparators, as well as orphan drug status, were significantly associated with G-BA's assessment outcomes (*P* = 0.018, *P* = 0.043, and *P* < 0.0001, respectively; Table 2). In addition, univariate analyses indicated that RCT design, low risk of bias, morbidity, and quality of life improvements also significantly influenced the outcomes (*P* < 0.0001, *P* = 0.001, *P* = 0.004, and *P* = 0.037, respectively; Supplementary Table S1).

**Table 2:** Multivariate logistic regression analysis of factors influencing G-BA's additional benefit assessment decisions.

**Variable**	**Coefficient**	**Standard error**	**95% Confidence intervals**	***P*-value**
**Bias risk (ref: high)**				
Low	−0.14	1.35	0.06–12.37	0.920
Not accessible	0.54	1.2	0.17–17.92	0.649
**RCT (ref: no)**				
Yes	−1.26	1.43	0.02–4.71	0.380
**Orphan drug (ref: no)**				
Yes	4.08	0.94	9.39–370.05	<0.0001^***^
**Mortality (ref: negative)**				
Positive	4.2	1.77	2.08–2,125.83	0.018^*^
No significant	−2.01	1.48	0.01–2.44	0.175
**Morbidity (ref: negative)**				
Positive	2.06	1.43	0.47–130.78	0.150
No significant	−0.29	1.37	0.05–11.07	0.833
Not accessible	−2.2	1.75	0–3.40	0.208
**QoL (ref: negative)**				
Positive	0.61	1.86	0.05–70.39	0.744
No significant	−1.82	1.51	0.01–3.16	0.230
Not accessible	0.31	1.57	0.06–29.87	0.844
**Side effects (ref:negative)**				
Positive	2.37	1.17	1.08–105.47	0.043^*^
No significant	−0.74	0.92	0.08–2.88	0.419
Not accessible	−5.21	1.87	0–0.21	0.005^**^
**Indication (ref: breast cancer)**				
Others	0.6	0.82	0.37–9.14	0.461
Public year	−0.32	0.20	0.49–1.07	0.102
Daily treatment cost average	0	0	1–1	0.680
Patient population average	0	0	1–1	0.916

This table reports the results of a multivariate logistic regression analysis, with the dependent variable being the G-BA assessment conclusion (whether additional benefit is recognized: additional benefit vs. no additional benefit).

The Coefficient in the table represents the regression coefficient β [i.e., log (OR)], the standard error denotes the standard error of β; the 95% Confidence Intervals represent the 95% confidence interval for the odds ratio (OR); the P-value indicates the result of a two-tailed test.

Categorical variables were coded using dummy variables, with ref denoting the reference group.

Significance levels: ^*^P <0.05, ^**^P <0.01, ^***^P <0.001.

**Figure 2:**
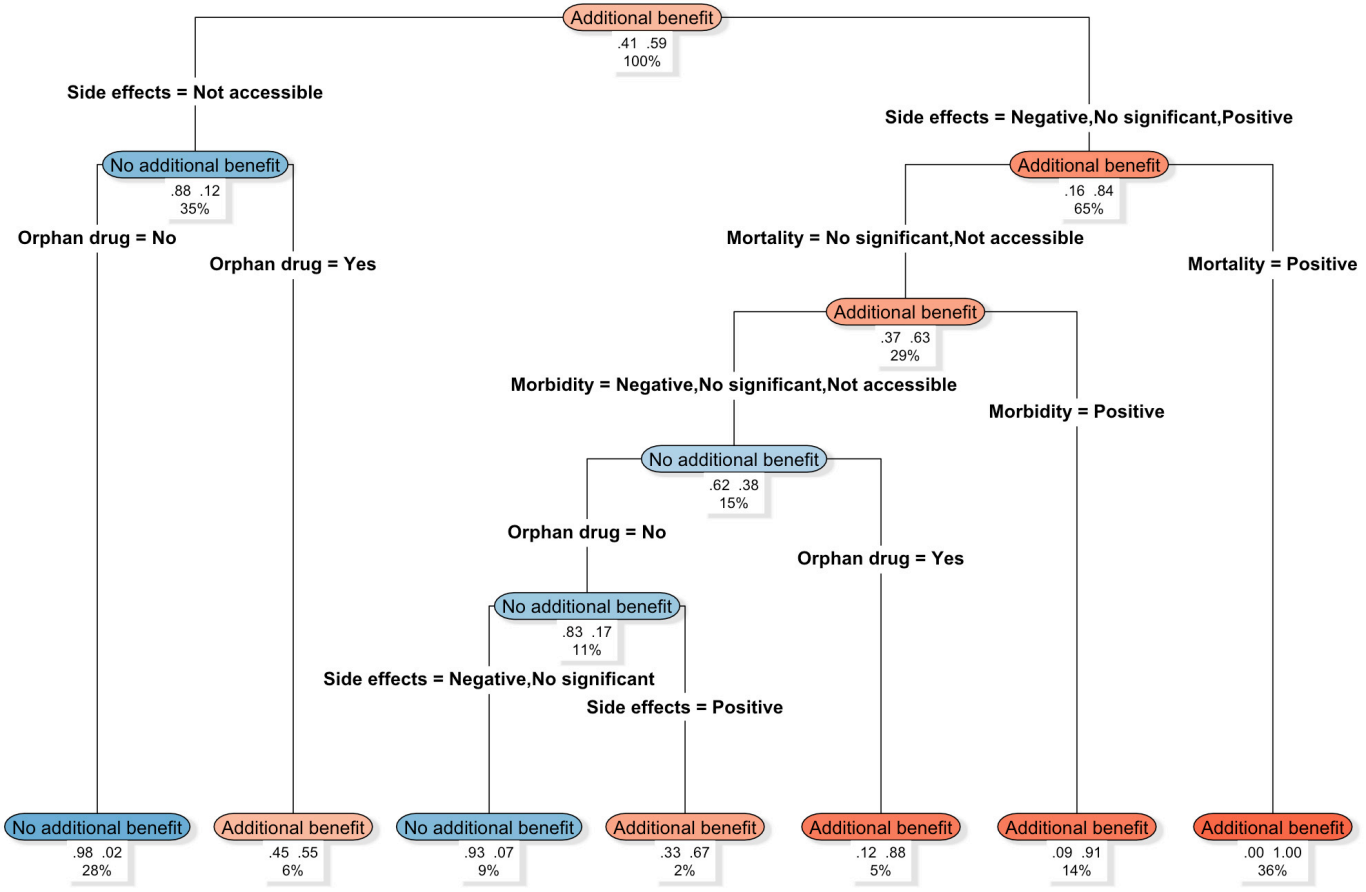
G-BA's decision pathway for additional benefit assessment. The model employs G-BA evaluation conclusions (Additional benefit, No additional benefit) as the dependent variable, incorporating variables such as Side effects, Mortality, Morbidity, and Orphan drug for pathway splitting. The decision tree displays the hierarchical influence of each variable on the additional benefit outcome from top to bottom. Each terminal node (from left to right) represents the proportion of “No additional benefit” vs. “Additional benefit” within that pathway. The percentage at the bottom indicates the proportion of this pathway within the entire sample. Node colors denote assessment outcome categories: orange signifies “Additional benefit”, blue indicates “No additional benefit”. Darker shades represent higher proportions of that outcome within the path. Variable descriptions: negative: worse outcomes compared to the control therapy. Positive: better outcomes or statistically significant improvement compared to the control therapy. No significant: no statistically significant difference observed compared to the control therapy. Not accessible: relevant outcome data was not reported or unavailable.

Using the rpart decision tree model, we classified the 171 G-BA assessments (Figure 2). Results indicate differing assessment tendencies across pathway combinations. Within this study sample, drugs were consistently assessed as “offering additional benefit” when adverse reaction data were available, and evidence demonstrated significant mortality improvement. Conversely, when adverse reaction data were unavailable, and the drug was non-orphan, the probability of being assessed as “no additional benefit” approached 98%. Furthermore, even in the absence of evidence demonstrating significant improvements in mortality or morbidity, drugs possessing orphan drug status still achieved an 88% probability of being assessed as “additional benefit”.

#### Key factors influencing the outcomes of NICE health technology assessments

The multivariate logistic regression indicated that risk of bias exerted significant effects on NICE outcomes. Compared with high-risk of bias studies, low risk of bias were significantly more likely to receive a positive recommendation (*P* = 0.028; Table 3). In univariate logistic regression analysis, compared with an ICER below £20,000/QALY, interventions with ICERs exceeding £30,000 per QALY were more likely to receive a negative NICE recommendation (*P* = 0.054; Supplementary Table S2).

**Table 3:** Multivariate logistic regression analysis of factors influencing NICE's health technology assessment decisions.

**Variable**	**Coefficient**	**Standard error**	**95% confidence intervals**	***P*-value**
**Bias risk (ref: high)**				
Low	1.68	0.76	1.20–23.77	0.028^*^
Medium	1.6	0.85	0.94–25.93	0.059
Not accessible	0.48	0.85	0.30–8.63	0.572
**RCT (ref: no)**				
Yes	0	0.75	0.23–4.37	0.998
**Orphan drug (ref: no)**				
Yes	−0.51	0.66	0.17–2.20	0.443
**OS (ref: no significant improvement)**				
Significant improvement	−0.69	0.85	0.10–2.63	0.413
Not accessible	−0.9	0.96	0.06–2.68	0.350
**PFS (ref: no significant improvement)**				
Significant improvement	−0.74	0.89	0.08–2.72	0.404
Not accessible	0.63	1.12	0.21–16.79	0.572
**QoL (ref: no significant improvement)**				
Significant improvement	0.36	1.18	0.14–14.42	0.758
Not accessible	0.17	0.81	0.24–5.83	0.833
**ICER (ref: £20,000 per QALY)**				
£20,000–£30,000 per QALY	−0.14	1.03	0.12–6.54	0.895
>30,000 per QALY	−1.63	0.97	0.03–1.32	0.094
Not accessible	−0.93	1.01	0.05–2.86	0.358
**Indication (ref: breast cancer)**				
Others	−0.17	0.6	0.26–2.75	0.780
Public year	−0.22	0.14	0.60–1.06	0.123
Daily treatment cost average	0	0	1–1	0.355

This table reports the results of a multivariate logistic regression analysis, with the dependent variable being the NICE assessment outcome (whether a recommendation was obtained: Recommended/Optimized vs. Not recommended).

The Coefficient represents the regression coefficient β [i.e., log (OR)], the Standard Error denotes the standard error of β.

The 95% Confidence Intervals represent the 95% confidence interval for the odds ratio (OR), and the P-value indicates the result of a two-tailed test.

All categorical variables were coded using dummy variables, with ref denoting the reference group.

Significance levels: ^*^P <0.05.

**Figure 3:**
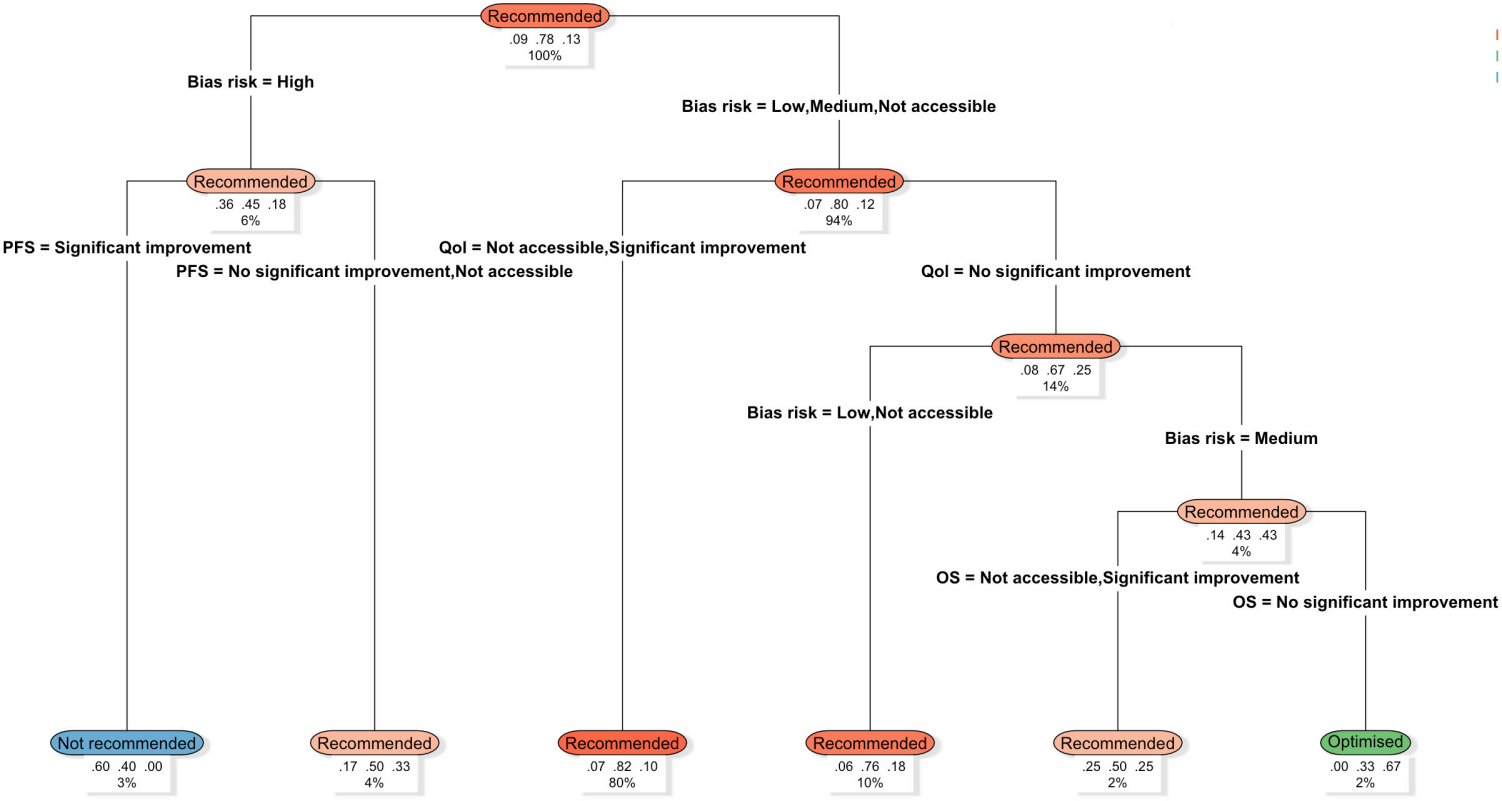
NICE's recommandation pathway for health technology assessment. The model employs NICE's assessment conclusions (Not recommended, Optimized, Recommended) as the dependent variable, incorporating clinical evidence variables such as bias risk, progression-free survival (PFS), overall survival (OS), and quality of life (QoL) for path splitting. The decision tree displays the hierarchical influence of each variable on the recommendation outcome in a top-down manner. The three sets of figures beneath each terminal node (left to right) represent the respective proportions for: not recommended, Optimized, and Recommended outcomes within that pathway. The bottom percentage indicates the proportion of the entire sample assigned to that pathway. Node colors denote assessment outcome categories: blue indicates not recommended, green denotes Optimized, and orange signifies Recommended. Darker shades represent higher proportions of that outcome within the path. Variable definitions: PFS, progression-free survival; OS, overall survival; QoL, quality of life; Not accessible: indicates the relevant outcome measure was unreported or unavailable.

Using a decision tree model, the outcomes of 171 NICE health technology assessments were categorized into pathways (Figure 3). For approximately 80% of drugs, when the risk of bias was low and quality of life improvements were evident, the probability of a positive recommendation reached 82%. Within the remaining 20% of cases, if the risk of bias was high and only progression-free survival (PFS) showed significant improvement, the probability of “not recommended” outcomes increased to 60%. Furthermore, when the risk of bias was moderate, and there was no significant improvement in either quality of life or overall survival (OS), the probability of receiving a conditional (optimized) recommendation reached 67%.

### Factors influencing the inconsistency between G-BA and NICE health technology assessment outcomes

For the 66 matched pairs exhibiting inconsistencies between G-BA and NICE assessments, a Firth-corrected logit regression model was employed to analyse factors influencing these discrepancies (Table 4). Results indicate that when clinical evidence is supported by RCTs, the likelihood of assessment inconsistency significantly decreases (OR = 0.24, *P* = 0.0003), suggesting that evidence grounded in RCTs enhances consistency between G-BA and NICE conclusions. Interaction term results indicated that the effect size of RCT evidence did not differ significantly between the two countries (*P* = 0.309), suggesting that RCT evidence generally influenced consistency in the same direction across both assessments.

**Table 4:** Firth logistic regression for factor × country (G-BA vs. NICE).

**Variable**	**Coefficient**	**Standard error**	**OR**	**95% Confidence intervals**	***P*-value**
**RCT**					
(Intercept)	0.67	0.36	1.96	0.98–4.12	0.057
RCT evidence	−1.42	0.41	0.24	0.11–0.52	0.0003^**^
Country (NICE vs. G-BA)	−0.48	0.48	0.62	0.24–1.58	0.319
RCT × Country	0.55	0.54	1.74	0.60–5.13	0.309
**Bias risk**					
(Intercept)	−0.82	0.19	0.44	0.30–0.64	<0.0001^***^
Bias risk	1.55	0.55	4.72	1.66–15.03	0.003^**^
Country (NICE vs. G-BA)	0.26	0.26	1.29	0.78–2.16	0.322
Bias risk × Country	−0.83	0.82	0.44	0.08–2.19	0.312
**Mortality**					
(Intercept)	−0.71	0.31	0.49	0.26–0.89	0.018^*^
Mortality	−1.47	0.52	0.23	0.08–0.61	0.003^**^
Country (NICE vs. G-BA)	0.39	0.47	1.48	0.58–3.78	0.406
Mortality × Country	1.08	0.67	2.95	0.81–11.51	0.103
**Morbidity**					
(Intercept)	−1.03	0.38	0.36	0.16–0.73	0.004^**^
Morbidity	−1.64	0.67	0.19	0.05–0.67	0.009^**^
Country (NICE vs. G-BA)	1.20	0.56	3.32	1.12–10.37	0.031^*^
Morbidity × Country	0.70	0.82	2.01	0.42–11.06	0.390

This table reports the results of a Firth penalized likelihood logistic regression analysis.

The dependent variable is the uniformly defined HTA assessment outcome (positive vs. non-positive), where G-BA's “Additional benefit” and NICE's “Recommended” are both classified as positive decisions.

The Country variable uses G-BA as the reference group (ref), with NICE denoting England's NICE assessment outcome.

Main effects indicate the association of factors within the G-BA evaluation framework; interaction terms (Factor × Country) denote the differential effect of NICE relative to G-BA.

Coefficient denotes the regression coefficient β [log (OR)], Standard Error represents the standard error of β, OR indicates the odds ratio [exp (β)], 95% Confidence Intervals denote the 95% confidence interval for OR, and P-value reflects the two-tailed test result.

Significance levels: ^*^P <0.05; ^**^P <0.01; ^***^P <0.001.

Further analysis revealed that improvements in mortality (OR = 0.23, *P* = 0.003) and morbidity (OR = 0.19, *P* = 0.009) similarly significantly reduced the probability of assessment inconsistency between the two countries. This suggests that improvements in key clinical endpoints help reduce divergence in assessment conclusions under different HTA frameworks. In contrast, lower study risk of bias showed a significant positive correlation with the probability of assessment inconsistency (OR = 4.72, *P* = 0.003), highlighting the critical role of bias risk in explaining G-BA and NICE assessment differences. Furthermore, statistically significant differences in national effects were observed only in the dimension of morbidity improvement (OR = 3.32, *P* = 0.031), with both G-BA and NICE HTA outcomes being influenced in the same direction by this dimension.

## Discussion

In this study, 171 matched drug-indication pairs were identified between G-BA and NICE. Compared with G-BA, NICE issued more favorable HTA outcomes. Specifically, 155 (90.64%) were recommended by NICE, whereas only 101 (59.06%) were evaluated by G-BA as providing additional benefit. Consistent with previous studies, the two institutions produced differing assessments for the same drug-indication matching pairs (24).

This divergence primarily reflects differences in the institutional positioning and decision-making functions of the two HTA systems. NICE recommendations integrate clinical efficacy, safety, and cost-effectiveness within the appraisal process and directly determine whether a drug is included in NHS reimbursement. In contrast, G-BA assessment of additional benefit is conducted at the stage of clinical value appraisal, with conclusions primarily informing subsequent price negotiations rather than constituting the final reimbursement decision. Consequently, the proportion of drugs deemed to provide additional benefit by G-BA is relatively low. However, this does not imply inferior reimbursement accessibility in Germany compared with England. Rather, it reflects Germany's phased decision-making framework, in which clinical value assessment precedes economic negotiation. Under this framework, drugs assessed as providing no additional benefit may still obtain reimbursement through the reference pricing system, albeit at restricted price levels.

Further analysis of the determinants of HTA outcomes and their decision pathways under the institutional frameworks of the G-BA and NICE revealed notable differences. Within the study sample, multivariate logistic regression showed that G-BA additional benefit determinations were significantly associated with improvements in mortality and adverse reactions relative to appropriate comparator therapies, as well as with orphan drug designation. Univariate logistic regression further indicated that randomized controlled trial (RCT) evidence, risk of bias, morbidity, and improvements in health-related quality of life were also significantly associated with G-BA assessment outcomes.

Consistent with Germany's current legal framework, this study empirically identified a significant association between orphan drug designation and G-BA determinations of additional benefit. Further examination of the sample indicates that some orphan drugs were granted an additional benefit rating despite the absence of critical endpoints, such as improvements in mortality or adverse reactions. This pattern is likely attributable to the specialized assessment pathway for orphan drugs within the German regulatory system. Under the AMNOG framework, orphan drugs with annual sales not exceeding.

€30 million may be directly recognized as providing additional benefit, with the G-BA assessing only their level of additional benefit.

Furthermore, the findings indicate that all-cause mortality is a critical factor influencing additional benefit assessments in Germany, which is consistent with the explicit prioritization of clinical endpoints in IQWiG's Allgemeine Methoden (25). Consequently, clinical trial designs should place greater emphasis on the completeness and statistical significance of all-cause mortality data. When such evidence is unavailable or fails to demonstrate statistically significant improvement, other robust evidence (e.g., patient-reported quality of life) becomes particularly important for substantiating the clinical value of an intervention.

Although previous studies have indicated that QoL plays a relatively limited role in additional benefit assessments, owing to factors such as inadequate data presentation or divergent interpretations of the concept between companies and regulatory bodies (26), we demonstrate that QoL nonetheless exerts a significant influence on additional benefit determinations. This discrepancy may be partly attributable to the characteristics of the sample included in this study and the analytical methods employed. The G-BA classifies QoL as one of the key clinical endpoints in assessing additional benefit. However, it should be emphasized that in particularly severe or life-threatening conditions, evidence of improvements in QoL alone is often insufficient to establish an additional benefit. If it cannot be adequately excluded that an intervention may have unacceptable adverse effects on serious morbidity or mortality, a positive benefit assessment cannot be concluded.

We found that the risk of bias exerts a statistically significant influence on NICE health technology assessment outcomes. Within our sample, studies with low to moderate risk of bias were associated with a substantially higher probability of receiving a favorable NICE recommendation (approximately 80%), underscoring the critical role of evidence quality in HTA decision-making. By contrast, even when statistically significant improvements in endpoints such as progression-free survival (PFS) were reported, interventions supported by evidence with a high risk of bias were more likely to receive unfavorable NICE recommendations.

Previous studies have indicated that cost-effectiveness analysis is the primary evaluation criterion used by NICE in its Health Technology Assessment (HTA) framework. Our findings are consistent with this observation. Notably, although variables such as RCT evidence, risk of bias, and morbidity did not emerge as statistically significant independent predictors in the multivariable model for NICE decisions in this study, this should not be interpreted as indicating a lesser emphasis on clinical efficacy or safety in NICE appraisals. Rather, NICE relies heavily on clinical evidence as a fundamental input to the construction of economic models and the estimation of health outcomes, which are subsequently synthesized into composite value metrics such as ICERs. Consequently, the influence of clinical evidence may be mediated through cost-effectiveness measures rather than appearing as an independent determinant in regression analyses. The observed divergence in decision-making between NICE and G-BA therefore primarily reflects differences in evaluation frameworks and policy objectives, rather than differences in the relative importance attached to clinical evidence.

An analysis of the 66 matched pairs in which G-BA and NICE produced divergent outcomes revealed that disagreements were less frequent when evaluations were based on high-quality clinical evidence, RCTs, low risk of bias, mortality improvement, and morbidity reduction. In such instances, the consistency between the two agencies was notably higher. This may be attributed to the capacity of high-quality evidence to mitigate the impact of differing evaluative frameworks and methodologies. Compared with QoL and other subjective measures, mortality and morbidity endpoints provide greater objectivity and reliability, thereby reducing the likelihood of interpretive bias and facilitating greater concordance between agencies. Existing research indicates that comparative clinical effectiveness is a key factor in HTA assessments. Both the G-BA and NICE explicitly state that direct comparative evidence based on appropriate comparator treatments should be prioritized as the foundation for effectiveness evaluations (27).

In the absence of head-to-head studies enabling direct comparison with an appropriate comparator therapy, indirect comparisons or non-randomized evidence may serve as supplementary sources of evidence. In German HTA practice, IQWiG places strong methodological emphasis on direct comparative evidence, sets stringent requirements for indirect comparisons, and typically does not accept non-randomized controlled data as sufficient efficacy justification (28). This characteristic is similarly reflected in the present study: RCT-based evidence was significantly associated with G-BA decisions, suggesting that direct comparative evidence against appropriate comparators is pivotal in assessing incremental benefits.

By contrast, within NICE's assessment framework, clinical evidence is integrated into economic models, and its influence is reflected through composite measures such as ICERs. Under this structure, the role of RCT evidence may be reflected indirectly through clinical outcome parameters and cost-effectiveness results, rather than appearing as an independent, statistically significant factor in multivariate models. Previous research suggests that NICE adopts a relatively more flexible approach to the acceptance of non-randomized evidence and indirect comparative studies (24, 28). However, these differences should be interpreted in light of divergent approaches to PICO specification and the selection of appropriate comparators across the two countries. Variations in the definition of interventions and comparators can affect the availability and structure of direct comparative evidence. Accordingly, observed differences in evidence formats or statistical significance should not be interpreted simplistically as reflecting differing standards of evidence, but rather as reflecting differences in decision-making structures and comparator definitions.

Overall, both Germany and England operate HTA systems that are internally coherent within their respective healthcare systems and payment frameworks, reflecting distinct institutional contexts and decision-making logics. G-BA centers on the classification of incremental benefits and links it to subsequent price-negotiation pathways. It establishes clear hierarchies and priorities for clinical endpoint assessment, enhancing the standardization of clinical value evaluation. Institutionally, it reinforces the binding role of clinical benefit classification outcomes in price negotiations (29). By contrast, NICE integrates clinical evidence with cost-effectiveness analysis into a unified decision-making framework. By translating clinical benefits into comparable value metrics through QALYs and ICERs, it enables payment decisions within explicit budget constraints, thereby facilitating access to innovative therapies within the limits of overall health system affordability. Within existing institutional frameworks, further incremental refinements may enhance decision transparency and operational feasibility. For G-BA, this could involve clarifying how incremental benefits at different levels are translated into specific price adjustments or discount mechanisms, thereby strengthening the predictability of price negotiations. For NICE, greater specification of how different types of clinical evidence are weighted within economic models, as well as clearer rules for handling associated uncertainties, could further improve the standardization of appraisal processes and the consistency of decision outcomes.

This study has several limitations. Firstly, it only included drug-indication pairs simultaneously assessed by both G-BA and NICE. Such drugs typically exhibit high costs or strong innovation, meaning the findings may pre-dominantly reflect characteristics of this category. Caution is required when extrapolating these results to HTA decisions concerning all cancer drugs. Secondly, in some NICE assessment reports, ICER values were either undisclosed or not finalized due to confidentiality agreements (such as Patient Access Schemes) or insufficient evidence. Although this study categorized such cases separately, the absence of ICER data may still affect the precise estimation of the role of cost-effectiveness variables. Thirdly, as NICE employs a lifetime time horizon and relies on model extrapolation, whereas G-BA prioritizes direct evidence from trial periods, this study's comparison of different outcome measures primarily reflects the differing decision-making mechanisms within distinct HTA frameworks. Finally, this study primarily focuses on institutional attributes and core assessment dimensions closely related to HTA for cancer drugs. Although these factors have been shown to be highly relevant in existing cross-national comparative research, they may still fail to encompass all institutional or contextual factors influencing HTA decisions.

## Conclusion

This study reveals that despite variations in assessment systems and frameworks across different countries, high-quality clinical evidence remains the core foundation for consistent decision-making. The findings hold significant value for optimizing drug reimbursement policies and enhancing the rational allocation of healthcare resources. Concurrently, they offer policymakers and enterprises important insights into the drivers of HTA decision-making. Furthermore, these conclusions may serve as a reference for companies formulating drug pricing negotiation and evidence submission strategies in Germany and England.

## Data Availability

The datasets presented in this study can be found in online repositories. The names of the repository/repositories and accession number(s) can be found in the article/Supplementary material.
