# Supplementary material for: A comparative analysis of health technology assessment decision-making for cancer drugs in Germany and England
Source: Front Public Health. 2026 Mar 17;14:1733239. doi: 10.3389/fpubh.2026.1733239 (PMC13035802; doi:10.3389/fpubh.2026.1733239)
Supplement: Supplementary file 1 [file Data_Sheet_1.docx]

# TableS1 Univariate Regression Analysis of Factors Influencing G-BA’s Additional benefit Assessment Decisions

**Variable Coefficient Bias risk (ref: High)**

**Standard Error**

**95% Confidence Intervals**

**P-value**

Low 1.84 0.55 2.17-18.46 **0.001****

Not accessible 0.15 0.65 0.32-4.19 0.818

**RCT (ref: No)**

| Yes | 1.61 | 0.42 | 2.20-11.33 | **<0.0001***** |
| --- | --- | --- | --- | --- |
| **Orphan drug (ref: No)** | | | | |
| Yes | 1.61 | 0.46 | 2.05-12.21 | **<0.0001***** |
| **Mortality (ref: Negative)** | | | | |
| Positive | 5.32 | 0.92 | 33.71-1227.31 | **<0.0001***** |
| No significant | 1.87 | 0.43 | 2.77-15.16 | **<0.0001***** |
| **Morbidity (ref: Negative)** | | | | |
| Positive | 1.76 | 1 | 0.82-41.42 | 0.078 |
| No significant | -0.09 | 0.89 | 0.16-5.23 | 0.920 |
| Not accessible | -2.47 | 0.87 | 0.02-0.46 | **0.004**** |
| **QoL (ref: Negative)** | | | | |
| Positive | 1.94 | 1.69 | 0.25-188.47 | 0.251 |
| No significant | -0.69 | 1.23 | 0.05-5.57 | 0.574 |
| Not accessible | -2.56 | 1.23 | 0.01-0.85 | **0.037*** |
| **Side effects (ref: Negative)** | | | | |
| Positive | 1.2 | 0.89 | 0.58-19.04 | 0.180 |
| No significant | -0.36 | 0.51 | 0.25-1.90 | 0.478 |
| Not accessible | -3.58 | 0.55 | 0.01-0.08 | **<0.0001***** |
| **Indication (ref: Breast cancer)** | | | | |
| Others | 0.17 | 0.35 | 0.6-2.35 | **0.623** |
| **Public year** | -0.15 | 0.08 | 0.74-1.01 | 0.058 |
| **Daily treatment cost average** | 0 | 0 | 1-1 | 0.455 |
| **Patient population average** | 0.0001 | 0.0001 | 1-1 | 0.173 |

**Note:** This table presents the results of univariate logistic regression analysis, with each variable entered into the model individually. The dependent variable is the G-BA assessment conclusion (whether additional benefit is recognised: Additional benefit vs No additional benefit). The Coefficient in the table represents the regression coefficient β (i.e., log(OR)), Standard Error denotes the standard error of β; 95% Confidence Intervals indicate the 95% confidence interval for the odds ratio (OR); P-values represent two-tailed test results. Categorical variables were coded using dummy variables, with ref denoting the reference group.

Significance levels: *P < 0.05; **P < 0.01; ***P < 0.001.

# TableS2 Univariate Regression Analysis of Factors Influencing NICE’s Health Technology Assessment Decisions

**Variable Coefficient Standard 95% Confidence P-value**

| **Error** | | | **Intervals** | |
| --- | --- | --- | --- | --- |
| **Bias risk (ref: High)** | | |  | |
| Low | 1.65 | 0.70 | 1.32-20.53 | **0.018^*^** |
| Medium | 1.53 | 0.78 | 1.00-21.23 | **0.050** |
| Not accessible | 0.95 | 0.79 | 0.56-12.12 | 0.225 |
| **RCT (ref: No)** | | | | |
| Yes | 0.09 | 0.58 | 0.35-3.38 | 0.880 |
| **Orphan drug (ref: No)** | | | | |
| Yes | -0.09 | 0.58 | 0.30-2.84 | 0.880 |
| **OS (ref: No significant improvement)** | | | | |
| Significant improvement | -0.98 | 0.79 | 0.08-1.76 | 0.215 |
| Not accessible | -0.71 | 0.81 | 0.10-2.41 | 0.380 |
| **PFS (ref: No significant improvement)** | | | | |
| Significant improvement | -0.86 | 0.81 | 0.09-2.07 | 0.289 |
| Not accessible | -0.11 | 0.87 | 0.16-4.94 | 0.903 |
| **QoL (ref: No significant improvement)** | | | | |
| Significant improvement | -0.3 | 1.08 | 0.09-6.10 | 0.779 |
| Not accessible | -0.07 | 0.69 | 0.24-3.61 | 0.919 |
| **ICER (ref: ≦20000)** | | | | |
| 20000-30000 | -0.37 | 0.97 | 0.10-4.62 | 0.703 |
| >30000 | -1.76 | 0.91 | 0.03-1.03 | **0.054** |
| Not accessible | -1.1 | 0.96 | 0.05-2.18 | 0.250 |
| **Indication (ref: Breast cancer)** | | | | |
| Others | 0.31 | 0.55 | 0.47-3.96 | 0.576 |
| **Public year** | -0.10 | 0.11 | 0.72-1.13 | 0.363 |
| **Daily treatment cost average** | 0 | 0 | 1-1 | 0.316 |

**Note:** This table presents the results of univariate logistic regression analysis, with each variable entered into the model individually. The Coefficient column denotes the regression coefficient β (i.e., log(OR)), Standard Error represents the standard error of β; 95% Confidence Intervals indicate the 95% confidence interval for the odds ratio (OR); P-values reflect two-tailed test results. Categorical variables were coded using dummy variables, with ref denoting the reference. Significance levels: *P < 0.05; **P < 0.01; ***P < 0.001.

# Decision Tree Model Parameter Specification

We employed the rpart package within R software to construct decision tree models, utilising the Classification and Regression Tree (CART) algorithm for classification analysis. Within the G-BA decision tree model, the dependent variable was defined as a binary classification outcome (Additional benefit vs No additional benefit); within the NICE decision tree model, the dependent variable was defined as a ternary classification outcome (Recommend, Not recommend, and Optimised). The model was configured with method = ‘class’, employing the Gini index as the node

splitting criterion. At each node, the variable maximising purity improvement was selected for splitting.

To control model complexity and prevent overfitting, the following stopping parameters were predefined: minimum split sample size (minsplit) set to 5, meaning further splitting is attempted only when a node contains at least 5 samples; minimum leaf node sample size (minbucket) set to 3, meaning terminal nodes must contain at least 3 observations. The complexity parameter (cp) is set to 0.001, meaning splits are retained only if the resulting model fit improvement exceeds this threshold; the maximum tree depth (maxdepth) is set to 8 to limit structural complexity. Model stability is assessed using 10-fold cross-validation (xval = 10).

The model underwent no post-pruning; instead, model complexity was controlled via the aforementioned predefined stopping rules. Variable importance was calculated based on the cumulative reduction in impurity across all splits. Decision tree analysis was conducted using the complete case data.

The CART algorithm was selected for its high interpretability, enabling the intuitive presentation of hierarchical splitting paths between variables. This facilitates the identification of key factors influencing health technology assessment decisions and their combinatorial patterns, while enhancing the reproducibility of analytical results.
